# KMT2A maintains stemness of gastric cancer cells through regulating Wnt/β-catenin signaling-activated transcriptional factor KLF11

**DOI:** 10.1515/med-2023-0764

**Published:** 2023-11-07

**Authors:** Chongwen Deng, Chunhua Ye, Xiwang Liao, Fuyin Zhou, Youxiong Shi, Hong Zhong, Junbiao Huang

**Affiliations:** Department of General Surgery, Loudi Central Hospital, No. 51, Changqing Middle Street, Loudi, 417000, People’s Republic of China; Department of General Surgery, Loudi Central Hospital, Loudi, 417000, People’s Republic of China

**Keywords:** gastric cancer, epigenesis, stemness, KMT2A, β-catenin, KLF11

## Abstract

The molecular mechanisms of epigenetic regulation in gastric cancer development are not yet well established. In this study, we demonstrated that KMT2A was highly expressed in gastric cancer and associated with poor outcomes of patients and revealed that KMT2A was significantly associated with stemness and increased nuclear β-catenin in gastric cancer. Mechanistically, KMT2A activated the translocation of β-catenin into the nucleus of gastric cancer cells, and then, β-catenin served as a coactivator of KLF11, which promoted the expression of specific gastric cancer stemness-related molecules, including SOX2 and FOXM1. Together, KMT2A is an important epigenetic regulator of gastric cancer stemness, which provides a novel insight to the potential application of targeting against KMT2A in treating gastric cancer.

## 1 Introduction

Gastric cancer is one of the common malignant tumors, which are threatening public health, with the second highest incidence and mortality among cancer patients in China. A large amount of evidence suggests that the development of gastric cancer is characterized by abnormal epigenetic modifications [1,2]. For example, the aberrantly activated Wnt/beta-catenin and P13K/Akt signaling pathways can be acetylated by histone H3 position 27 lysine residue (H3K27) modification to regulate epithelial–mesenchymal transition and promote the migration of gastric cancer cells [3]. Chromatin remodeling protein (such as MORC) repressed P21 gene transcription by recruiting histone deacetylase 1, which led to a significant increase in the number of cells in S-phase and G2/M-phase, and ultimately promoted gastric cancer cell proliferation [4]. In addition, E3 ubiquitin ligase is highly expressed in gastric cancer cells, and histone modifications regulated by it play an important role in the pathogenesis of gastric cancer [5]. Therefore, an in-depth study of abnormal alterations related to epigenetics may supply a scientific basis for understanding the pathogenesis of gastric cancer, which in turn provides new ideas for the prevention, early diagnosis, and treatment of gastric cancer.

In recent years, although many epigenetic progresses have made on gastric cancer, there are still many challenges to be faced. The exact molecular mechanisms of epigenetic regulation in various stages of gastric cancer development are not yet well established, and the interactions between various epigenetic modifications and their interactions with various genes and signaling pathways are unclear [6,7,8]. As it is well known, histone methylation is an important epigenetic modification that can occur at lysine or arginine residues and be catalyzed by histone methyltransferases. With the intensive research on various methyltransferases and the rapid development of molecular techniques, epigenetic modifications have become popular anti-cancer targets [9].

It has been demonstrated that histones maintain DNA structure, protect genetic information, and regulate gene expression. The amino-terminal (N-terminal) domain of histones extends out of the nucleosome and can interact with other regulatory proteins and DNA [10]. Modifications of histones include methylation, phosphorylation, acetylation, crotonylation, ubiquitination, glycosylation, and ADP-ribosylation. Imbalance of histone modifications can lead to tumor development, and loss of methylation and acetylation of histone H3 and H4 residues has been shown to be a marker of tumor cells [10,11]. Histone methylation is involved in the formation and maintenance of the heterochromatin structure, genomic imprinting, DNA repair, inactivation of X chromatin, and transcription, among other regulatory aspects [12]. Histone methylation regulates tumorigenesis, proliferation, metabolic reprogramming, epithelial–mesenchymal transition, invasion, and migration; meanwhile, histone methylation plays an important role in determining the efficacy and resistance of chemotherapy and targeted therapies in gastric cancer [13]. It is generally believed to be closely related to the genomic localization of methyltransferases and their enzymatic activity properties (product specificity) in catalyzing the generation of different methylation products. H3K4 methylation *in vivo* is mainly catalyzed by the histone-lysine N-methyltransferase 2 (KMT2) family of methyltransferases; in mammals, there are six KMT2 (MLL) family members, namely, KMT2A, MLL2, MLL3, MLL4, SET1A, and SET1B [14]. As the first-found member, KMT2A has recently been recognized to play an important role in gene dysregulation, cell malignant proliferation, and tumor cell growth and differentiation in cancers, while the KMT2A fusion protein produced by chromosomal translocation rearrangement is closely related to the development of cancers [15]. However, the role of KMT2A in gastric cancer is not well established. In the study, the prognostic value of KMT2A was demonstrated to be associated with poor outcome of gastric cancer patients and was recognized as a regulator of stemness in gastric cancer through regulating Wnt/β-catenin-induced expression of stemness-related genes. The findings will provide a solid foundation for the understanding of the association between KMT2A and stemness in gastric cancer.

## 2 Materials and methods

### 2.1 Clinical samples

To investigate the expression feature of KMT2A in gastric cancer, the public cancer database TCGA data (https://tcga⁃data.nci.nih.gov/tcga/) were first used, from which data on mRNA expression levels of KMT2A were downloaded in gastric cancer tissues (*n* = 408) and normal gastric tissues (*n* = 211); moreover, log2 [transcripts per million + 1] for log-scale to obtain expression data was used to produce box plots, as previous analysis procedure [16]. In addition, gastric cancer tissues and matched peritumor normal gastric tissues (*n* = 78, respectively) as well as 178 cancer samples that were subjected to paraffin embedding for further immunohistochemical examinations were acquired from patients undergoing surgical procedures. The diagnosis of gastric cancer was pathologically confirmed by three independent pathologists. A written consent was obtained from all of the patients.


**Ethical approval:** The study was approved by the Ethics Committee of the Loudi Central Hospital (Project ID: ECLCH/2021(03)).

### 2.2 Immunohistochemistry (IHC)

The formalin-fixed tissues were embedded in paraffin; sliced into 4 μm thick sections; and subsequently subjected to dewaxing, hydration, and antigen retrieval by heat; then, these sections were blocked with 5% goat serum for 30 min at room temperature and incubated overnight at 4°C with primary antibodies, which are as follows: anti-KMT2A (1:200; Merck, USA) and anti-CTNNB1 (1:100; CST, USA) diluted in 1× phosphate buffer saline (PBS) containing 2% bovine serum albumin. Then, the sections were subsequently washed three times with 1× PBS and incubated with horseradish peroxidase-conjugated goat anti-rabbit IgG secondary antibody, followed by incubation for 10 min with 3, 3′-diaminobenzidine tetrachloride after washing three times with 1× PBS and visualization of specific staining by light microscopy. Images were acquired under ×100 field with Leica MDi8 inverted microscope. Quantitative expression of immunostaining was carried out at a fixed threshold using ImageJ software (Maryland, USA).

### 2.3 Cell culture

Immortalized human BGC-823 cells were purchased from the National Collection of Authorized Cell Cultures (Chinese Academy of Sciences, Shanghai) for our study. Cell culture was performed according to the manufacturer’s protocol. BGC-823 cells were cultured in Dulbecco’s modified Eagle medium (DMEM) (HyClone, Thermo Fisher Scientific, Waltham, USA), supplemented with 10% (vol/vol) fetal bovine serum, 100 units/mL penicillin G, 100 μg/mL streptomycin sulfate, and 2 mM L-glutamine. All cells were cultured in humidified incubator under an atmosphere of 5% (vol/vol) CO_2_ in air at 37°C.

### 2.4 Lentivirus-mediated transfection

Lentivirus-mediated KMT2A knockdown and overexpression system and corresponding controls were constructed from GeneCreate (Wuhan, China). Transfection was performed using the transfection reagent Endo-Fectin^TM^ Max transfection (GeneCopoeia) according to the manufacturer’s protocols. Briefly, 50 nM of RNA oligonucleotides and 10 µL of Endo-Fectin^TM^ Max were diluted in 250 µL of Opti-MEM (Thermo Fisher Scientific, Inc.). Then, they were mixed and incubated at room temperature for 20 min to form a complex. Cells (1 × 10^5^) were incubated with the complex for 6 h, and then, the cells were maintained in fresh medium and harvested at indicated times after transfection for real-time polymerase chain reaction analysis and western blotting.

### 2.5 Cell viability

The cells were cultured in a 96‐well plate, with a density of 1 × 10^3^ cells/well. After treatment for the desired time, CCK‐8 kit (Dojindo, Japan) was used to detect the cell viability according to the manufacturer’s instructions. Multiskan GO microplate reader (Thermo Scientific, Waltham, MA, USA) was used to count the cells.

### 2.6 Quantitative reverse transcription PCR

Total RNA was extracted from cultured cell lines using TriZol (Invitrogen, USA) according to the manufacturer’s instructions. cDNA synthesis was conducted using a reverse transcription kit (FastQuant RT Kit; Tiangen, China). Real time-PCR was completed by SYBR qPCR Master Mix (Roche, USA) as recommended by the manufacturer and then followed by detection with an ABI 7500 (ABI, USA) and analyzed with the ABI SDS software (version 2.4, ABI, USA). The PCR primers are given in Table A1.

### 2.7 Western blotting

The proteins were extracted from gastric cancer cells by radio immunoprecipitation assay lysis buffer (Beyotime, China). Samples were loaded into 10% sodium dodecyl sulphate–polyacrylamide gel electrophoresis (SDS–PAGE), then electrophoresed, and transferred to a polyvinylidene fluoride membrane; after incubation with 5% skim milk, the membrane was incubated with primary anti‐KMT2A antibody (1:1,000; Merck, USA), anti-SOX2 antibody (1:1,000; CST, USA), anti-FOXM1 antibody (1:1,000; CST, USA), and anti‐glyceraldehyde-3-phosphate dehydrogenase (GAPDH) antibody (1:2,000; Abcam, USA) at 4°C overnight. After pouring off first antibodies, the membrane was rinsed briefly with tris buffered saline with tween buffer three times, 15 min for per time, and then, secondary antibody was added at appropriate dilution. Next, the membrane was rinsed with Tween‐20 three times, followed by incubation with horseradish peroxidase‐labeled goat‐anti‐rabbit IgG antibody (1:10,000; Abcam, USA) at 37°C. Blots were visualized using the SuperSignal West Pico Chemiluminescent Substrate (Thermo Fisher Scientific Inc.). Results are shown after the normalization of the loading amounts in each lane by GAPDH.

### 2.8 Cell apoptosis assay

The Annexin V-FITC/PI Apoptosis Detection Kit (#9124, AmyJet Scientific Inc. China) was used for detecting apoptotic cells by flow cytometry according to the manufacturer’s protocol. In brief, 5 × 10^5^ cells with different treatment were seeded in six-well plates and cultured for 36 h at 37°C. The adherent cells were separated out with trypsin–ethylene diamine tetraacetic acid. After centrifugation, the cells were washed twice with 1× PBS and then resuspended in 1× binding buffer. Subsequently, 100 μL of cell suspension was transferred to a culture tube and 5 μL of annexin V-FITC and 5 μL of propidium iodide buffer were added; after gentle mixing, the tube was incubated for 20 min at room temperature (20–25°C) in the dark. Before detection, 400 μL of 1× binding buffer was added to the culture tube. Fluorescence intensity was analyzed by flow cytometry.

### 2.9 Dual luciferase reporter assay

Luciferase Reporter Assay Kit ab287865 (previously known as Luciferase Reporter Assay Kit K801) provides a simple means for detecting luciferase activity in cells. Gastric cancer cells were transfected with the SOX2 or FOXM1 promoter reporter plasmids. For luciferase assay, 5 × 104 cells per well in 12-well plates were cultured without antibiotics overnight and then transfected with pGL3-SOX2/SOX2M or pGL3-FOXM1/FOXM1M and pcDNA3.1-KLF11. After 24 h, the cells were washed with PBS and subjected to lysis, and their luciferase activities were measured by using a dual luciferase assay kit (Promega). The results were normalized against Renilla luciferase. All transfections were performed in triplicate.

### 2.10 Chromatin immunoprecipitation-PCR assay

ChIP was performed as previously described [17]. Briefly, BGC-823 cells were harvested and cross-linked with 1% formaldehyde for 15 min at room temperature. Lysates were immunoprecipitated with Dynal magnetic beads and antibody against KLF11. DNA was isolated, and 10% immunoprecipitation (IP) lysate was used as input. Finally, ChIP products were amplified by PCR followed by electrophoretic analysis.

### 2.11 Co‐immunoprecipitation and mass spectrometry analysis (Co‐IP/MS)

Gastric cells in the 10 cm Corning dishes were lysed with IP lysis buffer (Beyotime), and the samples were placed on ice until flocculent turbidity appeared. After centrifugation at 16,900 g for 10 min, the supernatant was carefully removed and supplemented with anti-CTNNB1 antibody (1:50, CST, USA) at 4°C overnight. After centrifugation at 4,200 g for 2 min, the supernatant was carefully discarded and the remaining plaque was gently washed by IP lysis buffer three times. Then, a 50 μL of 1× loading buffer was added and vortexed at 100°C for 5 min. The sample was loaded into 10% SDS–PAGE, electrophoresed, and then visualized using a Fast Silver Staining Kit (Beyotime). Then the gel was cut, and the strips were analyzed using liquid chromatography tandem mass spectrometry (APEX IV, FT-MS, Bruker, USA).

### 2.12 Analysis of the methylation level of KLF11 promoter by methylation-specific polymerase chain reaction (MS-PCR)

DNA bisulfite modification was carried out using the EZ DNA Methylation-Direct kit (Zymo Research, USA) according to the manufacturer’s instructions. The bisulfite-treated DNA samples were then subjected to MS-PCR using primers for *KLF11* unmethylated sequence (forward 5′-TAGAGTTTTGTTTATGTGAGTGGTG-3′, reverse 5′-CTTATAAAAACCTCCTACAACCC C-3′) and for *KLF11* methylated sequence (forward 5′-TATTATAGAGTTTTGTTTATGTGA GTGT-3′, reverse 5′-CCCCTTATAAAAACCTCCTACACCACA-3′). The DNA products were amplified in 40 cycles at an annealing temperature of 60°C. Methylated PCR products (192 bp) were analyzed on 2% agarose gel.

### 2.13 Tumor sphere formation assay

Tumor sphere formation assay was performed as previously described [18]. BGC-823 cells were seeded into ultra-low attachment 24-well plates (Corning) in DMEM supplemented with 20 ng/mL of epidermal growth factor, 10 ng/mL of basic fibroblast growth factor (bFGF), 5 mg/mL of insulin, and 2% B27 at 37°C in a humidified atmosphere of 95% air and 5% CO_2_. Half medium with additions was renewed every other day. Tumorspheres were visualized and calculated by a microscope.

### 2.14 Statistical analysis

SPSS 24.0 software was used for the statistical analysis. Continuous variables were expressed as mean ± standard error of measurement (SEM) and compared using Wilcoxon test. Categorized variables were presented by frequency (*n*) and proportion (%) and then compared through analysis of variance. Independent *t*-test was used for the comparisons of the data between groups. Both Cox proportional hazard model and log‐rank test were applied to survival analyses. *P* < 0.05 was considered statistically significant.

## 3 Results

### 3.1 KMT2A is highly expressed in gastric cancer and associated with poor outcomes of patients

**Figure 1:**
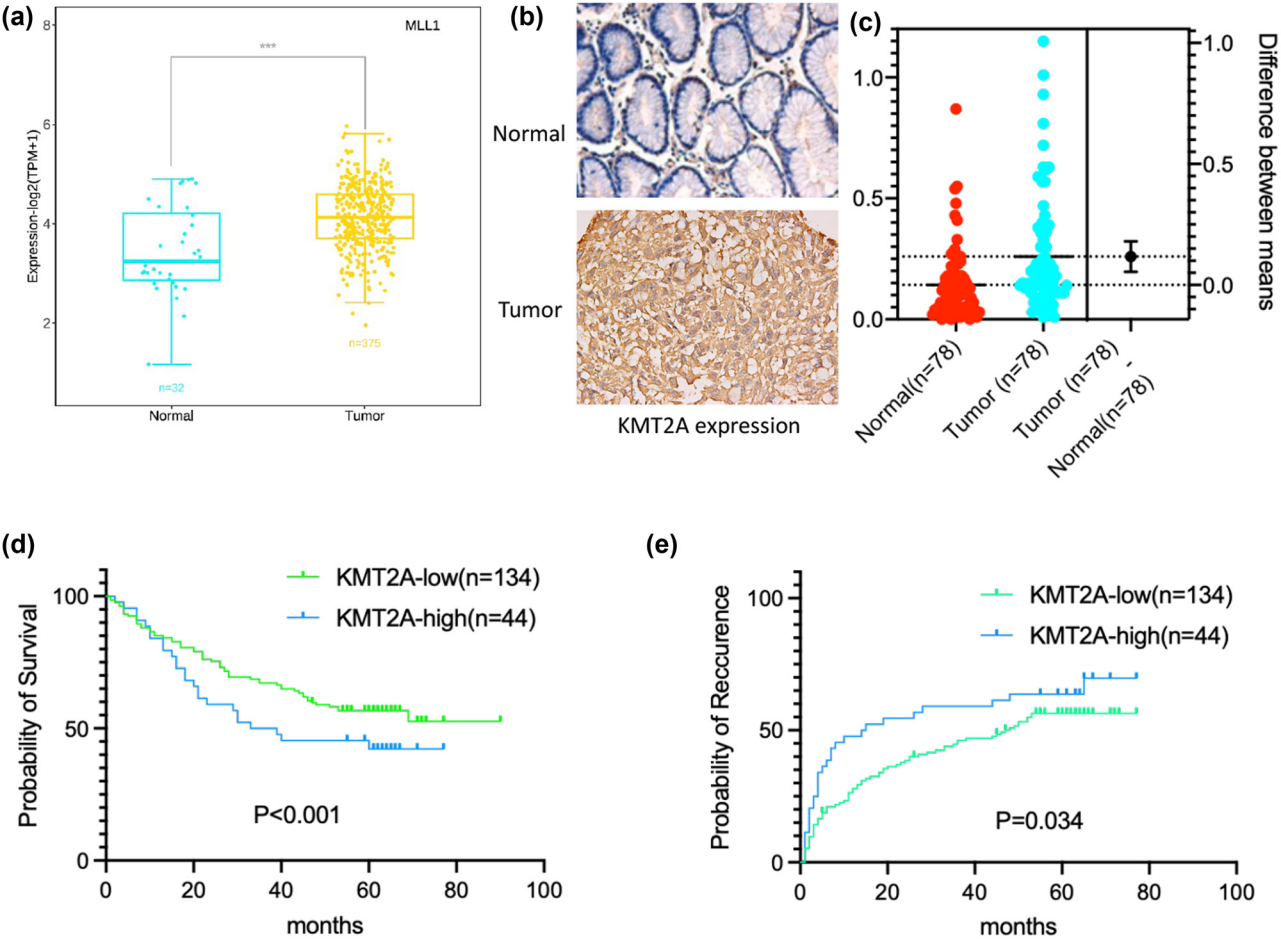
Expression and prognostic value of KMT2A in gastric cancer. (a) The expression of KMT2A in gastric cancer tissues and normal gastric tissues was analyzed based on data downloaded from the public cancer TCGA database (https://tcga⁃data.nci.nih.gov/tcga/). (b and c) KMT2A expression in collected gastric cancer tissues and matched peritumor normal gastric tissues using IHC assay. 200× magnification. (d and e) Kaplan–Meier estimates of OS time and recurrence in gastric cancer patients with different KMT2A expression.

To investigate the expression feature of KMT2A in gastric cancer, data were first downloaded from the public cancer database TCGA data (https://tcga⁃data.nci.nih.gov/tcga/) and preprocessed with mRNA expression RNASEqV2 data and KMT2A gene copy number for the gastric cancer dataset. The results of database analysis showed that the expression of KMT2A was significantly higher in gastric cancer tissues (*n* = 408) compared with that in normal gastric tissues (*n* = 211) (*P* < 0.01) (Figure 1a). Moreover, gastric cancer tissue and matched peritumor normal gastric tissue (*n* = 78, respectively) were collected and detected for KMT2A expression using IHC assay, and the result showed that KMT2A was highly expressed in cancer tissues, compared to matched normal tissues (*P* < 0.01), consistent with the result from TCGA dataset analysis (Figure 1b and c). Furthermore, independent risk factors for the prognosis of gastric cancer patients with KMT2A high expression, compared to those with KMT2A low expression, were assessed using COX logistic regression analysis and *log-rank* test in 178 gastric cancer tissues. The result showed that KMT2A was an independent risk factor for the prognosis of gastric cancer patients (Table 1). In addition, patient survival prognosis was analyzed using the *Kaplan–Meier* method. The results showed a significant correlation between expression level and prognosis (*P* < 0.001), with higher expression associated with worse prognosis (overall survival [OS] and progression-free survival [PFS]) (Figure 1d and e).

**Table 1:** COX logistic regression analysis on KMT2A expression in 177 patients with gastric cancer

Variates	*P* value	HR	95% CI
KMT2A (high vs low)	<0.001*	1.464	1.294–1.653
Age (>60 vs ≤60, ys)	0.524	1.184	0.778–1.765
Sex (male vs female)	0.997	1.009	0.698–1.432
T stage (T3 + T4 vs T1 + T2)	0.021*	1.725	1.082–2.739
N stage (N2 + N3 vs N1 + N0)	0.049*	1.529	0.981–2.179
M stage (M1 vs M0)	0.113	1.215	0.962–1.532

*P < 0.05.

### 3.2 KMT2A is significantly associated with stemness and increased nuclear β-catenin in gastric cancer

**Figure 2:**
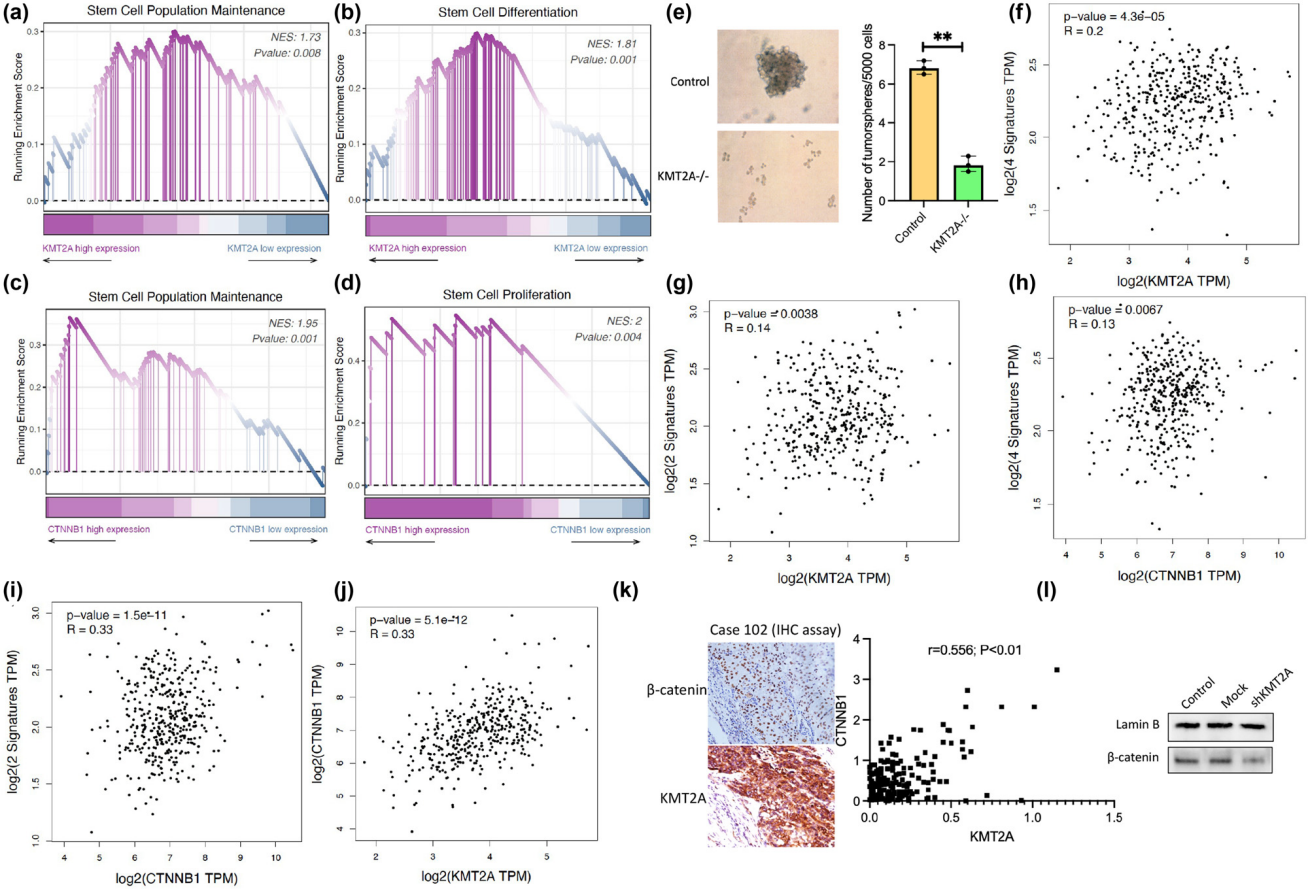
Correlation between KMT2A and stemness in gastric cancer patients and cells. (a and b) Correlation between KMT2A and stemness-related pathways in gastric cancer analyzed by GEPIA2 database. (c and d) Correlation between β-catenin and stemness-related pathways in gastric cancer. (e) Tumorsphere formation assay was performed in control and KMT2A^−/−^ BGC-823 cells. The number of tumorspheres means colonies per 5000 cells. (f–i) Correlation between KMT2A or β-catenin and gastric cancer stemness-related genes Sox2/FOXM1 and cancer stemness-related genes CD44/CD133/Sox2/OCT4, respectively, analyzed by the TIMER2.0 database. (j) Correlation between KMT2A and β-catenin expression in gastric cancer. (k) The expressions of KMT2A and β-catenin by IHC assay were analyzed using a Spearman method. (l) The nucleus extracts from BGC-823 cells with KMT2A knockdown, mock, and control were detected with anti-β-catenin antibody using a western blotting assay.

To screen for stemness-related signaling pathways associated with KMT2A expression in gastric cancer, we performed gene set enrichment analysis (GSEA) by comparing the high and low KMT2A expression groups. As shown in Figure 2a and b, stem cell differentiation-related pathways, including stem cell differentiation and stem cell population maintenance, were significantly enriched in gastric cancer with high expression of KMT2A. Moreover, to further investigate the relationship between KMT2A and stemness, we analyzed the correlation between KMT2A expression and the stemness-related marker set in gastric cancer using the GEPIA2 online web server. The result showed that a significant correlation was found between KMT2A and the stemness-related gene sets, including tumor stemness-related signature (CD44/CD133/Sox2/OCT4) and gastric cancer-specific stemness signature (Sox/FOXM1 [19]) (Figure 2c and d). In addition, to clarify the effect of KMT2A on the stemness of gastric cancer cells, we carried out a tumorsphere formation assay using BGC-823 cells. The result showed that the colony-forming ability of BGC-823 cells with KMT2A knockdown decreased significantly when compared to control cells (Figure 2e). Recent studies have demonstrated that the Wnt/β-catenin signaling pathway is involved in regulating the proliferation and differentiation of stem cells, closely related to the development of tumors and the tumor stem cell-induced drug resistance [20,21]. Hence, we further explored the correlation between β-catenin expression and stemness in gastric cancer. The result showed that in gastric cancer, high expression of β-catenin was positively associated with the regulation of stem cell differentiation and stem cell population maintenance; meanwhile, β-catenin expression showed a positive correlation with tumor stemness-related signature and gastric cancer-specific stemness signature (Figure 2f–i). Based on the findings, we further investigated the correlation between KMT2A expression and Wnt/β-catenin signaling in gastric cancer. The result showed a close association between KMT2A expression and β-catenin expression in gastric cancer (Figure 2j); moreover, the result from western blotting showed that also the high level of KMT2A protein was associated with high expression of β-catenin in clinical samples of gastric cancer (Figure 2k). In addition, it was also demonstrated that KMT2A knockdown inhibited the translocation of β-catenin to the nucleus in gastric cancer BGC-823 cells (Figure 2l).

### 3.3 KMT2A regulated stemness through modulating Wnt/β-catenin signaling in gastric cancer

**Figure 3:**
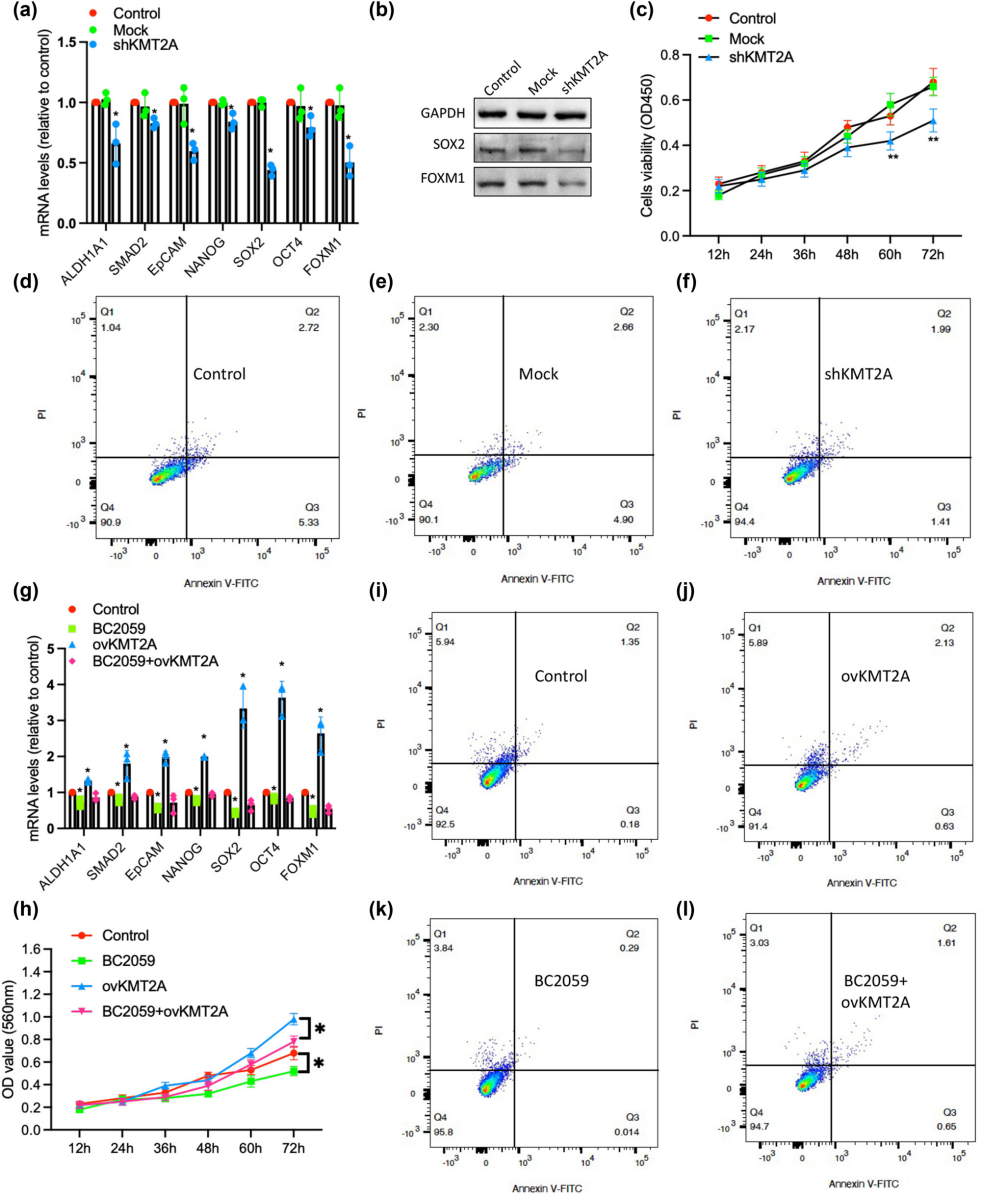
KMT2A regulated stemness through modulating Wnt/β-catenin signaling in gastric cancer. (a) mRNA levels of stemness-related factors, including ALDH1A1, SMAD2, EpCAM, NANOG, SOX2, OCT4, and FOXM1, in BGC-823 cells with KMT2A knockdown, mock, and control were determined by RT-PCR. (b) The protein levels of SOX2 and FOXM1 in BGC-823 cells with KMT2A knockdown, mock, and control were analyzed by a western blotting assay. (c) The cell proliferation was performed by CCK-8 assay. (d–f) The apoptosis of BGC-823cells with KMT2A knockdown, mock, and control was detected by a flow cytometry. Annexin V-FITC stands for the detecting the onset of apoptotic cells labeled with fluorescein isothiocyanate, and PI means cells with advanced apoptosis and loss of cell membrane integrity, showing red fluorescence; (g) mRNA levels of stemness-related factors in BGC-823 cells with KMT2A knockdown, BC2059 treatment, and combination were determined by RT-PCR. (h) The cell proliferation of BGC-823 cells with KMT2A knockdown, BC2059 treatment, and combination was determined using a CCK-8 assay. (i–l) The apoptosis of these cells was detected by a flow cytometry. Error bars indicate SEM; *n* = 3. *P < 0.05.

To further unravel the mechanism of KMT2A in affecting the stemness in gastric cancer, we observed the change of stemness-related factors and cell proliferation in BGC-823 cells with KMT2A knockdown, compared to control cells. The result indicated that the mRNA levels of some stemness-related factors, including ALDH1A1, SMAD2, EpCAM, NANOG, SOX2, OCT4, and FOXM1, were significantly decreased; meanwhile, the protein levels of gastric cancer-specific stemness molecules, including SOX2 and FOXM1, were also obviously reduced in BGC-823 cells with KMT2A knockdown (Figure 3a and b). Moreover, KMT2A knockdown inhibited significantly the proliferation of BGC-823 cells as well as promoted the apoptosis of these cells (Figure 3c and d). However, of note, when the nucleus translocation of β-catenin signaling was efficiently inhibited by Tegatrabetan (BC2059) treatment (100 nM; cat#,S0733, Selleck) in gastric cancer cells, KMT2A overexpression did not significantly upregulate the levels of these stemness-related molecules (Figure 3g) and rescue the proliferation (Figure 3h) as well as inhibit the apoptosis of gastric cancer cells (Figure 3i–l), compared to the KMT2A overexpression alone.

### 3.4 KMT2A modulates the expression of stemness-related genes through regulating β-catenin-activated KLF11 transcription in gastric cancer

**Figure 4:**
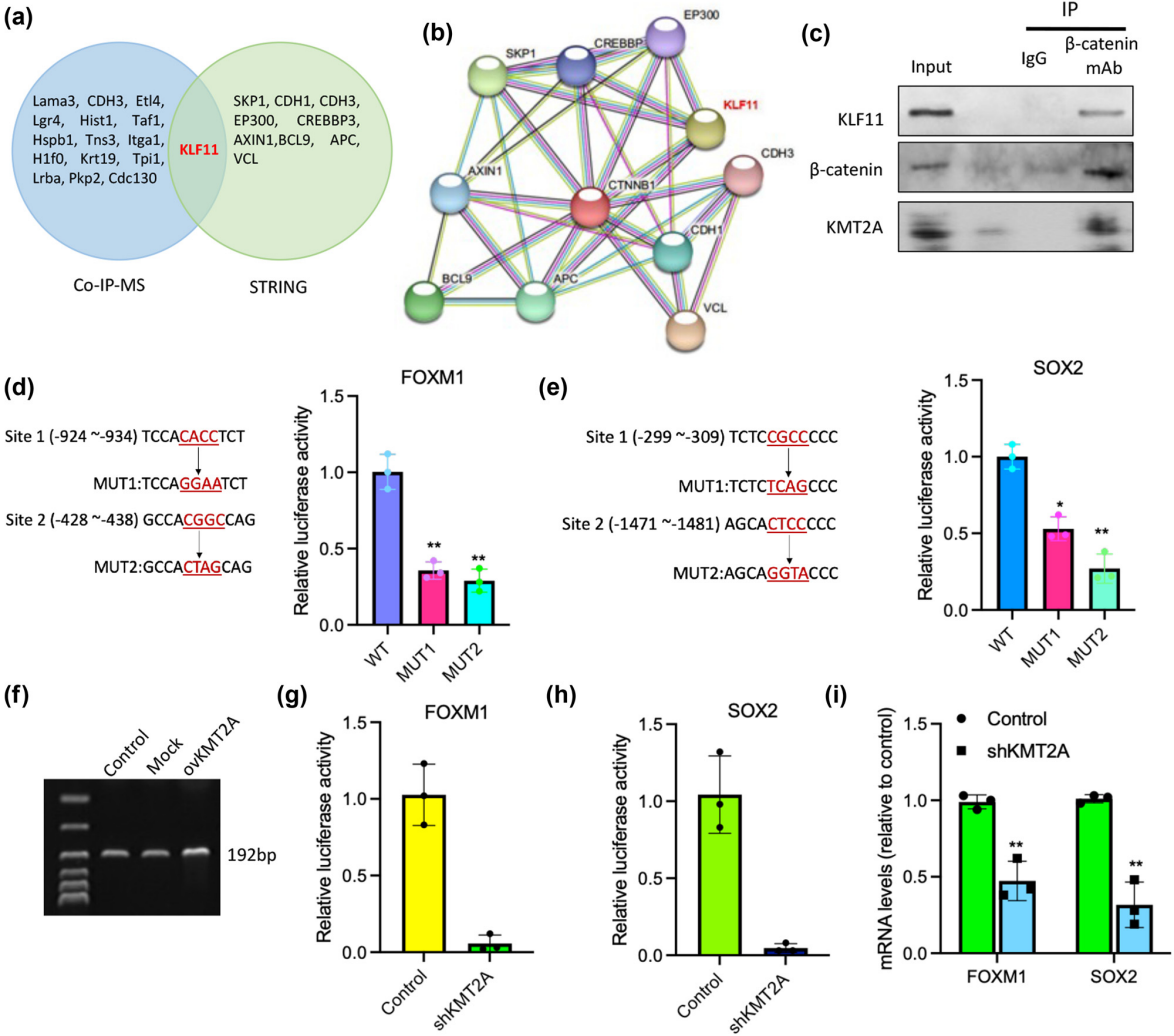
KMT2A-regulated stemness was dependent on KLF11 transcriptional activity. (a) The Venn diagram of β-catenin-binding nuclear proteins identified by Co-IP-MS and STRING prediction (b). (c) The immunoprecipitation by anti-β-catenin antibody was detected with anti-KLF11 or anti-KMT2A antibodies using a western blotting assay. (d and e) Predicted wild-type (WT) or mutated (Mut) full-length 3′-UTR of SOX2 and FOXM1 genes was cloned into a dual luciferase reporter plasmid and was then co-transfected with KLF11 vector; the activity was assessed by luciferase reporter gene assay. (f) MS-PCR analysis of KLF11 promoter in control BGC-823 cells, mock, and KMT2A-overexpressed BGC-823 cells. Methylated PCR products are 192 bp. (g–i) The luciferase activity of SOX2 and FOXM1 gene was determined by luciferase reporter gene assay. (h) The mRNA levels of SOX2 and FOXM1 were examined by a RT-PCR assay. Error bars indicate SEM; *n* = 3. *P < 0.05.

To further explore the molecular mechanism of KMT2A regulating β-catenin activation-induced stemness in gastric cancer, we identified the β-catenin-binding nuclear proteins by performing a co-immunoprecipitation/mass spectrometry (Co-IP/MS) analysis in BGC-823 cells. A total of 16 proteins were identified, namely, SKP1, CDH1, Etl4, Lgr4, Hist1, Taf1, Hspb1, Tns3, Klf11, Itga1, H1f0, Krt19, Tpi1, Vc1, Pkp2, and Cdc130 (Table A1). Meanwhile, among the nuclear proteins that can bind with β-catenin using STRING database, KLF11 was also found (Figure 4a and b). Hence, to clarify whether β-catenin can bind with KLF11 in gastric cancer cells, we carried out a Co-IP assay followed by western blotting detection. The result showed a binding of β-catenin with KLF11 in gastric cancer cells (Figure 4c). Moreover, it was identified by a dual luciferase reporter assay that KLF11 binds directly with the promoters of specific gastric cancer stemness-related molecules, including SOX2 and FOXM1 (Figure 4d and e). To unravel further the association between KMT2A and KLF11 expression, we first examined the output products of β-catenin Co-IP assay using the KMT2A antibody and found the existence of KMT2A in the β-catenin–KLF11 protein complex in BGC-823 cells (Figure 4c). Furthermore, it was also found that the methylation level of *KLF11* promoter was significantly increased in BGC-823 cells with KMT2A overexpression (Figure 4f). Of note, in gastric cells with KMT2A knockdown, KLF11 could not bind directly with the promoters of SOX2 and FOXM1 and initiate the transcription of SOX2 and FOXM1 (Figure 4g–i).

## 4 Discussion

Epigenetic alteration has been recognized as the important feature of cancers [22]. A comprehensive understanding of the characteristic alterations in epigenetic modifications during tumor development will facilitate the development of novel combination therapeutic strategies for tumors. In recent decades, targeting epigenetic regulation of histones or DNA for tumor therapy has attracted increasing attention [23]. Histone lysine methyltransferases play a variety of important roles in the maintenance of chromatin stability and the regulation of gene expression by catalyzing the transfer of methyl groups to specific lysine side chains at the H3 and H4 ends of histones to form histone methylation characteristics, thereby affecting gene transcription, DNA replication, and DNA repair [24]. Histone lysine methyltransferases have been reported to be closely associated with the occurrence of various cancers and diseases [25], and their potential to be used as therapeutic targets is unlimited. Recently, as a lysine methyltransferase, KMT2A has also been found to act as a transcriptional cofactor that plays an important role in regulating gene expression during early development and hematopoiesis [26,27]. However, the roles of KMT2A in solid cancers are not still clear. More recently, KMT2A has been recognized as a novel and potentially predictive biomarker for immune checkpoint inhibitor therapy in a variety of solid tumors [28]. In the study, we investigated the expression feature of KMT2A in gastric cancer through analyzing the public cancer TCGA dataset and detecting the clinical samples and found the significantly higher expression of KMT2A in gastric cancer; then, the prognostic value of KMT2A was further assessed, and it was demonstrated that high expression of KMT2A was associated with worse prognosis (OS and PFS). The results suggest that KMT2A could be used as a poor prognostic factor, and highly expressed KMT2A was significantly associated with shorter survival and risk of recurrence in gastric cancer patients. It is the first time to unravel the prognostic value of KMT2A expression, but not mutation, in gastric cancer, although KMT2A mutations, including *KMT2A*-PTD, have been demonstrated as a potential predictor in other cancer, particularly in leukemia [29].

Recent advances have highlighted the involvement of epigenetic deregulation in cancer metastasis, stemness, and drug resistance [30]; meanwhile, the dysregulation of lysine methyltransferases has been recognized as a notable epigenetic trait in gastric cancer [31]. Recently, it is reported that KMT2A functions as an epigenetic regulator of cancer stemness in intestinal cancer [32]. To define the association between KMT2A expression and stemness of gastric cancer, we performed GSEA of stemness-related signaling pathways and explored the correlation between KMT2A expression and a stemness-related marker set. It was found that stem cell differentiation-related pathways, including stem cell differentiation and stem cell population maintenance, were significantly enriched in gastric cancer with high expression of KMT2A; meanwhile, a significant correlation was observed between KMT2A expression and the stemness-related gene sets, including tumor stemness-related signature (CD44/CD133/Sox2/OCT4) and gastric cancer-specific stemness signature (Sox/FOXM1). Furthermore, we identified a critical role of KMT2A in promoting the stemness formation of gastric cancer cells using a tumorsphere formation assay. These results suggested a strong relationship between KMT2A expression and stemness in gastric cancer. Hence, it is necessary to further explore the mechanism by which KMT2A is involved in the stemness regulation of gastric cancer.

As is well known, the WNT signaling pathway is an ancient and evolutionarily conserved signaling system that regulates the self-renewal balance between embryonic development and adult cells by relying not only on local effector elements but also on β-catenin to recruit several chromosomal modifiers to form specific functional and structural regions of the chromosome [20]. It has recently been found that WNT signaling regulates histone acetyltransferase activity, leading to widespread structural changes in chromosomes [33]; moreover, WNT/β-catenin signaling can also regulate the EMX-dependent stemness in sarcoma [34]. To explore the correlation between β-catenin expression and stemness in gastric cancer, we demonstrated that high expression of β-catenin was positively association with the regulation of stem cell differentiation, stem cell population maintenance, and the signatures of tumor stemness-related signature and gastric cancer-specific stemness. Meanwhile, a close association between KMT2A expression and β-catenin expression in gastric cancer was also observed; moreover, KMT2A regulated the translocation of β-catenin to the nucleus in gastric cancer cells. The findings indicated the involvement of KMT2A in gastric cancer stemness by activating the WNT/β-catenin signaling. Additionally, in the study, it was also demonstrated that when the nucleus translocation of β-catenin signaling was efficiently inhibited by Tegatrabetan (BC2059) treatment in gastric cancer cells, KMT2A overexpression did not significantly upregulate the levels of these stemness-related molecules and rescue the proliferation as well as inhibit the apoptosis of gastric cancer cells. Altogether, the results suggested that KMT2A-induced stemness was dependent on the activation of Wnt/β-catenin signaling in gastric cancer cells. Based on our findings, a close correlation between Wnt/β-catenin signaling, epigenetic regulation, and stemness in cancers has been unraveled, which is consistent with recent advances in function of Wnt signaling-related epigenetic regulation/stemness [35,36].

To address the issue on the molecular mechanism of KMT2A regulating β-catenin activation-induced stemness in gastric cancer, we identified the β-catenin-binding nuclear protein KLF11 by performing a Co-IP/MS analysis gastric cancer cells. KLF11, a member of the Kruppel-like transcription factor family, is involved in regulating cell proliferation, cell cycle, and apoptosis [37]. Studies have shown that KLF11 is closely related to the development of tumors [38,39,40], and further in-depth research can better understand the mechanisms of KLF11 involved in tumorigenesis and development. Hence, in this study, we further demonstrated KLF11 binds directly with the promoters of SOX2 and FOXM1, but could not initiate the transcription of SOX2 and FOXM1 in gastric cells with the inhibition of β-catenin translocation to the nucleus or KMT2A knockdown. Meanwhile, KMT2A did not only directly bind to β-catenin–KFL11 complex but also affect the methylation of KFL11 promoter, finally regulating the expression of FKL11 in gastric cancer cells. All of the aforementioned results suggested at the first time that KMT2A activated the translocation of β-catenin into the nucleus of gastric cancer cells and then served as a coactivator of KLF11, which promoted the expression of specific gastric cancer stemness-related molecules, including SOX2 and FOXM1.

In conclusion, we demonstrated the effects of KMT2A on stemness of gastric cancer. Mechanistically, KMT2A activated β-catenin signaling, which served as a coactivator of KLF11, and promoted the expression of specific gastric cancer stemness-related molecules. This study provides a novel insight to the potential application of targeting against KMT2A as an epigenetic inhibitor. Nevertheless, further studies would be required to elucidate the specific epigenetic regulatory mechanism in gastric cancer in the future.

## Appendix

**Table A1:** Primers used for real time PCR and shRNA sequences

Genes	Forward (5′−3′)	Reverse (5′−3′)
GAPDH	GCA CCG TCA AGG CTG AGA AC	TGG TGA AGA CGC CAG TGG A
SOX2	GAC AGT TAC GCG CAC ATG AA	ACA CAG CTG GGT GGA AGA GA
FOXM1	GGGCGCACGGCGGAAGATGAA	CCACTCTTCCAAGGGAGGGCTC
ALDH1A1	ACACTGCAACAGGAGGACCAAGAA	ACATGCCCAATGACCTCACCT
SMAD2	CCTGTTGTGACTGTGGATGGCTATG	AGACCTTTATATACGCGCTTGGGTAGA
EpCAM	GAAGGCTGAGATAAAGGAGATGGG	TTAACGATGGAGTCCAAGTTCTGG
OCT4	GGT ATT CAG CCA AACGA CCA	CAC ACT CGG ACC ACA TCC TT
NANOG	GTG ATT TGT GGG CCTGA AGA	ACA CAG CTG GGT GGA AGA GA
CTNNB1	CTATGCAGGGGTGGTCAACA	CTGGAAAACGCCATCACCAC
KMT2A shRNA	CCGGGCACTGTTAAACATTCCACTTCTCGAGAAGTGGAATGTTTAACAGTGCTTTTTG	AATTCAAAAAGCACTGTTAAACATTCCACTTCTCGAGAAGTGGAATGTTTAACAGTGC

**Table A2:** Proteins that bind β-catenin in nuclear fractions of gastric cancer cells

Gene symbol	Annotation	Mean relative abundance	Median Preimmune Ratio	*P*(*x* ^2^)	Nucleus?
Skp1	S-Phase Kinase Associated Protein 1	670	∞	0.0001	Yes
Cdh1	Cadherin-1	502	∞	0.0001	Yes
Etl4	Sickle tail protein homolog	339	∞	0.0001	Yes
Lgr4	Leucine-rich repeat-containing G-protein coupled receptor 4	132	∞	0.0001	Yes
Hist1	Histone H4	88	∞	0.0001	Yes
Taf1	Transcription initiation factor TFIID subunit 1	62	∞	0.0001	Yes
Hspb1	60 kda heat shock protein, mitochondrial	32	∞	0.0029	Yes
Tns3	Tensin-3	22	∞	0.0032	Yes
Klf11	KLF Transcription Factor 11	14	∞	0.0209	Yes
Itga1	Integrin alpha-1	9	∞	0.0392	Yes
H1f0	Histone H1.0	5	∞	0.0002	Yes
Krt19	Keratin, type I cytoskeletal 19	3	∞	0.0021	Yes
Tpi1	Triosephosphate isomerase	63	8.92	0.0045	Yes
Vcl	Vinculin	141	6.21	0.0325	Yes
Pkp2	Plakophilin 2	204	3.25	0.0023	Yes
Cdc130	Coiled-coil domain-containing protein 130	103	1.62	0.0236	Yes

## References

[1] Ebrahimi V, Soleimanian A, Ebrahimi T, Azargun R, Yazdani P, Eyvazi S, et al. Epigenetic modifications in gastric cancer: Focus on DNA methylation. Gene. 2020;742:144577. 10.1016/j.gene.2020.144577 32171825

[2] Fattahi S, Amjadi-Moheb F, Tabaripour R, Ashrafi GH, Akhavan-Niaki H. PI3K/AKT/mTOR signaling in gastric cancer: Epigenetics and beyond. Life Sci. 2020;262:118513. 10.1016/j.lfs.2020.118513 33011222

[3] Cui H, Hu Y, Guo D, Zhang A, Gu Y, Zhang S, et al. DNA methyltransferase 3A isoform b contributes to repressing E-cadherin through cooperation of DNA methylation and H3K27/H3K9 methylation in EMT-related metastasis of gastric cancer. Oncogene. 2018;37(32):4358–71. 10.1038/s41388-018-0285-1 PMC6085280 29717263

[4] Singh A, Chang TY, Kaur N, Hsu KC, Yen Y, Lin TE, et al. CAP rigidification of MS-275 and chidamide leads to enhanced antiproliferative effects mediated through HDAC1, 2 and tubulin polymerization inhibition. Eur J Med Chem. 2021;215:113169. 10.1016/j.ejmech.2021.113169 33588178

[5] Yao Y, Liu Z, Huang S, Huang C, Cao Y, Li L, et al. The E3 ubiquitin ligase, FBXW5, promotes the migration and invasion of gastric cancer through the dysregulation of the Hippo pathway. Cell Death Discov. 2022;8(1):79. 10.1038/s41420-022-00868-y PMC8873275 35210431

[6] Patel TN, Roy S, Ravi R. Gastric cancer and related epigenetic alterations. Ecancermedicalscience. 2017;11:714. 10.3332/ecancer.2017.714 PMC5243136 28144288

[7] Padmanabhan N, Ushijima T, Tan P. How to stomach an epigenetic insult: the gastric cancer epigenome. Nat Rev Gastroenterol Hepatol. 2017;14(8):467–78. 10.1038/nrgastro.2017.53 28513632

[8] Tang SY, Zhou PJ, Meng Y, Zeng FR, Deng GT. Gastric cancer: An epigenetic view. World J Gastrointest Oncol. 2022;14(1):90–109. 10.4251/wjgo.v14.i1.90 PMC8790429 35116105

[9] Grady WM, Yu M, Markowitz SD. Epigenetic alterations in the gastrointestinal tract: current and emerging use for biomarkers of cancer. Gastroenterology. 2021;160(3):690–709. 10.1053/j.gastro.2020.09.058 PMC7878343 33279516

[10] Choi SJ, Jung SW, Huh S, Chung YS, Cho H, Kang H. Alteration of DNA methylation in gastric cancer with chemotherapy. J Microbiol Biotechnol. 2017;27(8):1367–78. 10.4014/jmb.1704.04035 28621113

[11] Rahman Z, Bazaz MR, Devabattula G, Khan MA, Godugu C. Targeting H3K9 methyltransferase G9a and its related molecule GLP as a potential therapeutic strategy for cancer. J Biochem Mol Toxicol. 2021;35(3):e22674. 10.1002/jbt.22674 33283949

[12] Wang F, Zhao J, Liu D, Zhao T, Lu Z, Zhu L, et al. Capsaicin reactivates hMOF in gastric cancer cells and induces cell growth inhibition. Cancer Biol Ther. 2016;17(11):1117–25. 10.1080/15384047.2016.1235654 PMC5137488 27715462

[13] Chen Y, Ren B, Yang J, Wang H, Yang G, Xu R, et al. The role of histone methylation in the development of digestive cancers: a potential direction for cancer management. Signal Transduct Target Ther. 2020;5(1):143. 10.1038/s41392-020-00252-1 PMC7398912 32747629

[14] Li Y, Zhao L, Zhang Y, Wu P, Xu Y, Mencius J, et al. Structural basis for product specificities of MLL family methyltransferases. Mol Cell. 2022;82(20):3810–25.e8. 10.1016/j.molcel.2022.08.022 36108631

[15] Lin W, Francis JM, Li H, Gao X, Pedamallu CS, Ernst P, et al. Kmt2a cooperates with menin to suppress tumorigenesis in mouse pancreatic islets. Cancer Biol Ther. 2016;17(12):1274–81. 10.1080/15384047.2016.1250986 PMC5199165 27801610

[16] Wang J, Yuan Y, Tang L, Zhai H, Zhang D, Duan L, et al. Long Non-Coding RNA-TMPO-AS1 as ceRNA Binding to let-7c-5p Upregulates STRIP2 Expression and Predicts Poor Prognosis in Lung Adenocarcinoma. Front Oncol. 2022;12:921200. 10.3389/fonc.2022.921200 PMC9237420 35774125

[17] Tong D, Zhang J, Wang X, Li Q, Liu L, Lu A, et al. MiR-22, regulated by MeCP2, suppresses gastric cancer cell proliferation by inducing a deficiency in endogenous S-adenosylmethionine. Oncogenesis. 2020;9(11):99. 10.1038/s41389-020-00281-z PMC7652948 33168819

[18] Johnson S, Chen H, Lo PK. In vitro tumorsphere formation assays. Bio Protoc. 2013;3(3):e325. 10.21769/bioprotoc.325 PMC4972326 27500184

[19] Wei C, Chen M, Deng W, Bie L, Ma Y, Zhang C, et al. Characterization of gastric cancer stem-like molecular features, immune and pharmacogenomic landscapes. Brief Bioinform. 2022;23(1):bbab386. 10.1093/bib/bbab386 34571533

[20] Zhan T, Rindtorff N, Boutros M. Wnt signaling in cancer. Oncogene. 2017;36(11):1461–73. 10.1038/onc.2016.304 PMC5357762 27617575

[21] Xu X, Zhang M, Xu F, Jiang S. Wnt signaling in breast cancer: biological mechanisms, challenges and opportunities. Mol Cancer. 2020;19(1):165. 10.1186/s12943-020-01276-5 PMC7686704 33234169

[22] Zhu L, Yang X, Zhu R, Yu L. Identifying discriminative biological function features and rules for cancer-related long non-coding RNAs. Front Genet. 2020;11:598773. 10.3389/fgene.2020.598773 PMC7772407 33391350

[23] Hogg SJ, Beavis PA, Dawson MA, Johnstone RW. Targeting the epigenetic regulation of antitumour immunity. Nat Rev Drug Discov. 2020;19(11):776–800. 10.1038/s41573-020-0077-5 32929243

[24] Black JC, Van Rechem C, Whetstine JR. Histone lysine methylation dynamics: establishment, regulation, and biological impact. Mol Cell. 2012;48(4):491–507. 10.1016/j.molcel.2012.11.006 PMC3861058 23200123

[25] Husmann D, Gozani O. Histone lysine methyltransferases in biology and disease. Nat Struct Mol Biol. 2019;26(10):880–9. 10.1038/s41594-019-0298-7 PMC6951022 31582846

[26] Zhao S, Allis CD, Wang GG. The language of chromatin modification in human cancers. Nat Rev Cancer. 2021;21(7):413–30. 10.1038/s41568-021-00357-x PMC10507815 34002060

[27] Rao RC, Dou Y. Hijacked in cancer: the KMT2 (MLL) family of methyltransferases. Nat Rev Cancer. 2015;15(6):334–46. 10.1038/nrc3929 PMC4493861 25998713

[28] Zhang R, Wu HX, Xu M, Xie X. KMT2A/C mutations function as a potential predictive biomarker for immunotherapy in solid tumors. Biomark Res. 2020;8(1):71. 10.1186/s40364-020-00241-0 PMC7724704 33298164

[29] Ye W, Ma M, Wu X, Deng J, Liu X, Zheng X, et al. Prognostic significance of KMT2A- PTD in patients with acute myeloid leukaemia: a systematic review and meta-analysis. BMJ Open. 2023;13(2):e062376. 10.1136/bmjopen-2022-062376 PMC9896228 36725100

[30] Nachiyappan A, Gupta N, Taneja R. EHMT1/EHMT2 in EMT, cancer stemness and drug resistance: emerging evidence and mechanisms. FEBS J. 2022;289(5):1329–51. 10.1111/febs.16334 34954891

[31] Norollahi SE, Mansour-Ghanaei F, Joukar F, Ghadarjani S, Mojtahedi K, Gharaei Nejad K, et al. Therapeutic approach of Cancer stem cells (CSCs) in gastric adenocarcinoma; DNA methyltransferases enzymes in cancer targeted therapy. Biomed Pharmacother. 2019;115:108958. 10.1016/j.biopha.2019.108958 31075731

[32] Grinat J, Heuberger J, Vidal RO, Goveas N, Kosel F, Berenguer-Llergo A, et al. The epigenetic regulator Mll1 is required for Wnt-driven intestinal tumorigenesis and cancer stemness. Nat Commun. 2020;11(1):6422. 10.1038/s41467-020-20222-z PMC7752919 33349639

[33] Mosimann C, Hausmann G, Basler K. Beta-catenin hits chromatin: regulation of Wnt target gene activation. Nat Rev Mol Cell Biol. 2009;10(4):276–86. 10.1038/nrm2654 19305417

[34] Jimenez-Garcia MP, Lucena-Cacace A, Otero-Albiol D, Carnero A. Empty spiracles homeobox genes EMX1 and EMX2 regulate WNT pathway activation in sarcomagenesis. J Exp Clin Cancer Res. 2021;40(1):247. 10.1186/s13046-021-02048-9 PMC8348834 34364391

[35] Amjadi-Moheb F, Akhavan-Niaki H. Wnt signaling pathway in osteoporosis: Epigenetic regulation, interaction with other signaling pathways, and therapeutic promises. J Cell Physiol. 2019;234(9):14641–50. 10.1002/jcp.28207 30693508

[36] Teeuwssen M, Fodde R. Wnt Signaling in Ovarian Cancer Stemness, EMT, and Therapy Resistance. J Clin Med. 2019;8(10):1658. 10.3390/jcm8101658 PMC6832489 31614568

[37] Lin L, Mahner S, Jeschke U, Hester A. The distinct roles of transcriptional factor KLF11 in normal cell growth regulation and cancer as a mediator of TGF-beta signaling pathway. Int J Mol Sci. 2020;21(8):2928. 10.3390/ijms21082928 PMC7215894 32331236

[38] Ji Q, Li Y, Zhao Q, Fan LQ, Tan BB, Zhang ZD, Zhao XF, et al. KLF11 promotes gastric cancer invasion and migration by increasing Twist1 expression. Neoplasma. 2019;66(1):92–100. 10.4149/neo_2018_180325N201 30509092

[39] Wang Y, Wu J, Chen H, Yang Y, Xiao C, Yi X, et al. Genome-wide CRISPR-Cas9 screen identified KLF11 as a druggable suppressor for sarcoma cancer stem cells. Sci Adv. 2021;7(5):eabe3445. 10.1126/sciadv.abe3445 PMC7840125 33571129

[40] Ellenrieder V, Buck A, Harth A, Jungert K, Buchholz M, Adler G, et al. KLF11 mediates a critical mechanism in TGF-beta signaling that is inactivated by Erk-MAPK in pancreatic cancer cells. Gastroenterology. 2004;127(2):607–20. 10.1053/j.gastro.2004.05.018 15300592

